# Arginine Vasopressin Plays a Role in Microvascular Dysfunction After ST‐Elevation Myocardial Infarction

**DOI:** 10.1161/JAHA.123.030473

**Published:** 2023-09-08

**Authors:** Ayman Al‐Atta, Luke Spray, Ashfaq Mohammed, Evgeniya Shmeleva, Ioakim Spyridopoulos

**Affiliations:** ^1^ Freeman Hospital Newcastle upon Tyne United Kingdom; ^2^ Translational and Clinical Research Institute, Vascular Biology and Medicine Theme, Faculty of Medical Sciences Newcastle University Newcastle Upon Tyne United Kingdom; ^3^ Tufts University Boston MA USA

**Keywords:** acute myocardial infarction, copeptin, microvascular dysfunction, microvascular obstruction, Percutaneous Coronary Intervention, Acute Coronary Syndromes, Coronary Artery Disease

## Abstract

**Background:**

Coronary microvascular dysfunction (CMD) predicts mortality after ST‐elevation–myocardial infarction (STEMI). Arginine vasopressin (AVP) may be implicated, but data in humans are lacking, and no study has investigated the link between arginine vasopressin and invasive measures of CMD.

**Methods and Results:**

We invasively assessed CMD in 55 patients with STEMI treated with primary percutaneous coronary intervention (PPCI), by measuring the index of microcirculatory resistance after PPCI. In a separate group of 45 patients with STEMI/PPCI, recruited for a clinical trial, we measured infarct size and microvascular obstruction with cardiac magnetic resonance (CMR) imaging at 1 week and 12 weeks post‐STEMI. Serum copeptin was measured at 4 time points before and after PPCI in all patients with STEMI. Plasma copeptin levels fell from 92.5 pmol/L before reperfusion to 6.4 pmol/L at 24 hours. Copeptin inversely correlated with diastolic, but not systolic, blood pressure (r=−0.431, *P*=0.001), suggesting it is released in response to myocardial ischemia. Persistently raised copeptin at 24 hours was correlated with higher index of microcirculatory resistance (r=0.372, *P*=0.011). Patients with microvascular obstruction on early CMR imaging showed a trend toward higher admission copeptin, which was not statistically significant. Copeptin levels were not associated with infarct size on either early or late CMR.

**Conclusions:**

Patients with CMD after STEMI have persistently elevated copeptin at 24 hours, suggesting arginine vasopressin may contribute to microvascular dysfunction. Arginine vasopressin receptor antagonists may represent a novel therapeutic option in patients with STEMI and CMD.

**Nonstandard Abbreviations and Acronyms**
AVP: arginine vasopressin
CFR: coronary flow reserve
CMD: coronary microvascular dysfunction
IMR: index of microcirculatory resistance
MVO: microvascular obstruction

**Clinical Perspective**

**What Is New?**

- After ST‐segment–elevation myocardial infarction, persistently elevated copeptin, a stable plasma surrogate of the vasoactive hormone arginine vasopressin, is associated with invasive measures of coronary microvascular dysfunction.

**What Are the Clinical Implications?**

- Measurement of copeptin may identify patients at risk of coronary microvascular dysfunction, an adverse prognostic finding after ST‐segment–elevation myocardial infarction, and allow targeted therapies.
- Arginine vasopressin antagonists may represent a novel therapeutic strategy in patients with ST‐segment–elevation myocardial infarction, aiming to improve the function of the coronary microcirculation.

Primary percutaneous coronary angiography (PPCI) is the gold standard of care for patients presenting with ST‐segment–elevation myocardial infarction (STEMI).^1^, ^2^ Despite routine use of PPCI and optimal medical therapies, long‐term outcomes after STEMI remain poor, with heart failure incidence of approximately 20% at 5 years.^3^ PPCI successfully restores epicardial blood flow in 95% of patients, but as few as 50% achieve complete reperfusion of the distal coronary vasculature.^4^ This is broadly termed coronary microvascular dysfunction (CMD) and is associated with adverse outcomes including heart failure and mortality.^5^

CMD may be seen during invasive coronary angiography as the no‐reflow phenomenon, where relief of the epicardial occlusion does not lead to distal flow of contrast. Angiographic assessment, however, is limited by the subjectivity of common angiographic scores, such as myocardial blush grade and Thrombolysis In Myocardial Infarction perfusion grade.^6^ CMD also manifests as microvascular obstruction (MVO) and intramyocardial hemorrhage on cardiac magnetic resonance (CMR) imaging, and CMR‐detected CMD is a significant predictor of cardiac death, reinfarction, heart failure, and stroke even after adjusting for infarct size.^7^ However, routine implementation of CMR in post‐STEMI care is limited by availability, cost, and the lack of therapeutic options specifically targeting CMD.

The index of microcirculatory resistance (IMR), measured during invasive angiography, more directly and quantitatively assesses coronary microvascular function. IMR is a thermodilution‐based method that detects the minimum resistance of the microcirculation, measured during maximal hyperemia.^8^ IMR has greater reproducibility than other angiographic measures of CMD and predicts CMR‐determined infarct size, MVO, and intramyocardial hemorrhage.^9^ An IMR value >40 at the end of PPCI is an independent predictor of mortality at 1 to 2 years follow‐up,^10^, ^11^, ^12^ and several studies have investigated IMR‐directed therapies with limited success.^13^ Despite these promising advances, IMR currently remains rare outside of clinical research. Novel, accessible biomarkers of CMD after STEMI are needed, both for risk stratification and the discovery of new therapeutic targets to relieve CMD.

Arginine vasopressin (AVP) is a hormone synthesized in the hypothalamus and stored in the posterior pituitary gland. AVP regulates water homeostasis and blood pressure, and its release is triggered by changes in extracellular volume and physiological stress.^14^ In both animal and human studies, AVP is immediately released after MI.^15^, ^16^ The effect of AVP on coronary vascular tone is controversial, but evidence from animal models suggests a reduction in coronary blood flow due to microvascular vasoconstriction,^17^ suggesting it may mediate CMD after STEMI. AVP is unstable in plasma, so copeptin, the C‐terminal part of preprovasopressin, which is cosecreted with AVP in equimolar amounts and stable in plasma, is commonly used as a surrogate marker for AVP release.^18^

The relationship of copeptin to infarct size has been reported in a small number of studies, with conflicting results.^19^, ^20^, ^21^ To our knowledge, no previous study has investigated a link between copeptin concentration and either invasive or noninvasive measures of CMD. In this study, we hypothesize that greater AVP release, detected by higher copeptin concentrations, will predict CMD in patients with STEMI and may represent a novel therapeutic target.

## METHODS

### Data Availability Statement

The raw data that support the findings of this study will be available from the corresponding author upon reasonable request.

### Patient Populations

This study used 2 cohorts of patients. The MRI cohort was composed of 45 patients with STEMI recruited to the single‐center, randomized, double‐blinded, placebo‐controlled CAPRI (Evaluating the Effectiveness of Intravenous Ciclosporin on Reducing Reperfusion Injury in Patients Undergoing Primary Percutaneous Coronary Intervention) trial (NCT02390674). We also prospectively recruited 55 additional patients with STEMI, in whom invasive CMD assessment with IMR and CFR was performed in the culprit artery at the end of the PPCI procedure (the IMR cohort). All participants were recruited between March 2015 and April 2020. Baseline characteristics of the included patients are displayed in Table 1. Inclusion and exclusion criteria were similar between STEMI cohorts and are shown in Table 2.

**Table 1:** Demographic Details of the Study Cohorts

	All patients n=100	IMR cohort (n=55)				MRI cohort (n=45)
		All	IMR ≤40 (n=32)†	IMR >40 (n=22)	*P* value	
Age, y	62.0±1.1	59.5±1.4	57.4±1.9	62.2±1.9	0.09	65.0±1.6
Male sex	82 (82.0)	44 (80.0)	26 (81.3)	18 (81.8)	0.96	38 (84.4)
White race	…	53 (98.1)	31 (96.9)	22 (100)	1.00	…
Body mass index, kg/m^2^	28.7±0.7	29.7±1.0	29.0±1.3	31.0±1.6	0.33	28.2±0.7
Estimated glomerular filtration rate, mL/min per 1.73 m^2^	85.4±2.1	84.0±2.1	85.3±3.0	81.7±3.2	0.42	87.1±3.8
Diabetes	9 (9.1)*	6 (11.1)*	4 (12.5)	2 (9.5)	0.74	3 (6.7)
Hypertension	25 (25.3)*	19 (35.2)*	12 (37.5)	7 (33.3)	0.46	6 (13.3)
Hypercholesterolemia	10 (10.1)*	6 (10.9)	5 (15.6)	1 (4.5)	0.20	4 (8.9)
Current smoker	29 (29.6)*	18 (34.0)*	12 (37.5)	6 (30.0)	0.17	11 (24.4)
Family history	41 (44.1)*	24 (50.0)*	13 (48.1)	11 (55.0)	0.64	17 (37.8)
Peak troponin, ng/L	3439±285	2949±343	2370±410	3855±577	0.04	4168±459
Anterior infarct	49 (49.0)	20 (36.4)	12 (37.5)	7 (31.8)	0.67	14 (31.1)
Onset‐to‐reperfusion time, min	209.4±11.2	222.2±16.9	209.2±23.7	245.4±24.0	0.30	193.7 (13.6)
Stent length, mm	33 (23–48)	33 (23–44)	33 (23–48)	33 (28–38)	0.56	36 (25–57)
Stent diameter, mm	3.5 (3.1–3.9)	3.5 (3.5–4.0)	3.5 (3.5–4.0)	3.5 (3.0–4.0)	0.74	3.5 (3.0–3.5)
Glycoprotein inhibitors IIb/IIIa used	64 (64.7)*	34 (61.8)	18 (56.3)	15 (68.2)	0.38	30 (68.2)*
Aspiration thrombectomy	30 (30.0)	19 (34.5)	11 (34.4)	7 (31.8)	0.85	11 (24.4)

The IMR cohort was split into low and high IMR. *P* values were determined with independent samples *t* tests for continuous variables, which are presented as mean±SEM, Mann–Whitney *U* tests for continuous variables, which are presented as median (interquartile range), and chi‐square tests for binary variables, which are presented as n (%). IMR indicates index of microcirculatory resistance; and MRI indicates magnetic resonance imaging.

* Some missing values.

^†^ IMR could not be measured in 1 patient.

**Table 2:** Inclusion and Exclusion Criteria for the Study Population

Inclusion criteria	Exclusion criteria
(1) Patients presenting with acute myocardial infarction (ST‐segment–elevation) <12 h and undergoing percutaneous coronary intervention (primary PCI). (2) Age≥18 y (3) Not involved in any other research projects (4) Pre‐PPCI TIMI flow of 0/I (5) Able to understand and read English (6) Willing and able to provide verbal informed consent (7) Post PCI TIMI flow II or III	(1) Clinically unstable patients (hemodynamically unstable, shocked, unconscious patients) (2) Previous myocardial infarction (3) Patients lack capacity (4) Contraindication to adenosine (severe asthma) (5) Post‐PCI TIMI flow 0 or I (6) Inability to obtain index of microcirculatory resistance (7) History of coronary artery bypass graft (8) Out‐of‐hospital cardiac arrest unless resuscitated and stable

PPCI indicates primary percutaneous coronary intervention; STEMI, myocardial infarction; and TIMI, thrombolysis in myocardial infarction.

### Measurement of Copeptin and Other Hormones

In all cohorts, serial blood samples were drawn for copeptin analysis. They were collected in EDTA tubes and centrifuged for 15 minutes at 1500*g* within 1 hour of collection. Plasma was stored at −80 °C until analysis. Plasma copeptin was determined using a commercially available automated immuno‐florescent assay (BRAHMS Copeptin proAVP) run on a Kryptor Compact Plus analyzer (BRAHMS/Thermo Scientific, Henningsdorf, Germany). The measuring range of this assay is 0.7 to 2000 pmol/L. Baseline copeptin was measured before reperfusion in both STEMI cohorts. In the IMR cohort, we then measured copeptin at 30 minutes, 180 minutes, and 24 hours after reperfusion. In the MRI cohort, we measured copeptin at 30 minutes, 90 minutes, and 24 hours after reperfusion. The researcher performing copeptin assays was blinded to the IMR and MVO data linked to the samples.

### MRI Protocol

CMR scans were performed at day 2 to 7 as well as at 12 weeks following STEMI using a Siemens Avanto 1.5 Tesla MRI scanner with a phased array body coil combined with a spine coil. Gadobutrol contrast (Gadovist, Bayer Schering Pharma AG, Berlin, Germany) was administered intravenously in a dose of 0.1 mmol/kg, and short axis end‐diastolic late gadolinium enhancement images were obtained 10 minutes later. CMR data were analyzed using a validated software (cvi42, Circle Cardiovascular Imaging Inc., Calgary, Canada) as previously described.^22^, ^23^ MVO and infarct size were calculated as a percentage of left ventricular mass.

### IMR Protocol

In the IMR cohort, we measured IMR and CFR in the culprit vessel at the end of PPCI using a thermodilution technique. A standard pressure wire (Pressure Wire X Guidewire, Abbott) was calibrated, equalized, and advanced to the distal one third of the culprit vessel. Then, 200 μg of intracoronary glyceryl trinitrate was administered, and the following parameters were measured both at baseline and after hyperemia was induced with intravenous infusion of adenosine at a rate of 140 to 180 μg/kg per minute: (1) mean aortic pressure, (2) mean distal coronary pressure, and (3) mean transit time. Mean transit time was calculated as the average of 3 transit time measurements during 3 separate intracoronary injections of 3 mL of room temperature 0.9% saline solution. IMR was then calculated as mean distal pressure at hyperemia multiplied by mean transit time at hyperemia. CFR is calculated by dividing the resting mean transit time by the hyperemic mean transit time. Previous work has linked an IMR of ≥40 to adverse outcomes after STEMI,^13^ so this was used to define patients with high or low IMR values.

### PPCI Procedure

PPCI was performed as per local hospital protocol, and the use of dual antiplatelet therapies, anticoagulation, and aspiration atherectomy was at the discretion of the operators.

### Study Approval

For the IMR cohort, Health Research Authority and Health Care Research Wales approval was obtained; REC Reference 20/NE/0094. The CAPRI study received a favorable ethical opinion from the National Research Ethics Committee North‐East—Newcastle and North Tyneside 2 (REC reference: 14/NE/1070). All patients gave written, informed consent to participate. Patients with STEMI initially gave witnessed, verbal consent on arrival to hospital, with written consent gained at the earliest opportunity after PPCI.

### Power Calculations

The prospectively recruited IMR cohort was powered to identify a significant difference in 24‐hour copeptin concentration between patients with low (≤40) and high (>40) IMR. To identify a difference of 11.5 pg/mL (SD 10 pg/mL), with 80% power and a significance level of 5%, 15 patients in each group were required. By recruiting a larger number of patients, we have even greater power in this comparison, and accounted for difficulty in predicting the IMR of recruited patients. The sample size for the MRI cohort, however, was not determined by power calculation, as these patients had all been recruited for the CAPRI trial and we retrospectively analyzed cryopreserved blood samples. We measured AVP, and other hormones, in a subgroup of 13 patients, a sample powered to find changes in AVP. Previous evidence suggests that AVP increases by 10 pg/mL (SD 8 pg/mL) after acute MI.^16^ To detect this difference, with 80% power and a significance level of 5%, a sample size of 10 is required. We have used a larger cohort to improve power, especially in measuring other hormones for which fewer preliminary data are available.

### Statistical Analysis

Statistical analysis was performed with SPSS version 28, and graphs were produced with GraphPad Prism version 9. Hypothesis testing was with the independent *t* test for normally distributed data or the Mann–Whitney *U* test for nonnormally distributed data, including copeptin values. Admission copeptin was not normally distributed (Shapiro–Wilk <0.001), so correlations between copeptin and other values were assessed with Spearman's rho.

## RESULTS

### Demographic Characteristics of the Study Population

This study included a total of 100 patients. The mean age of the whole group is 62 years, and men represented 82% of the included population. A total of 49% of the patients had an anterior MI. Baseline and procedural characteristics were not different between the groups (Table 1).

### Copeptin Is Released After Myocardial Infarction and Normalizes by 24 Hours

**Figure 1:**
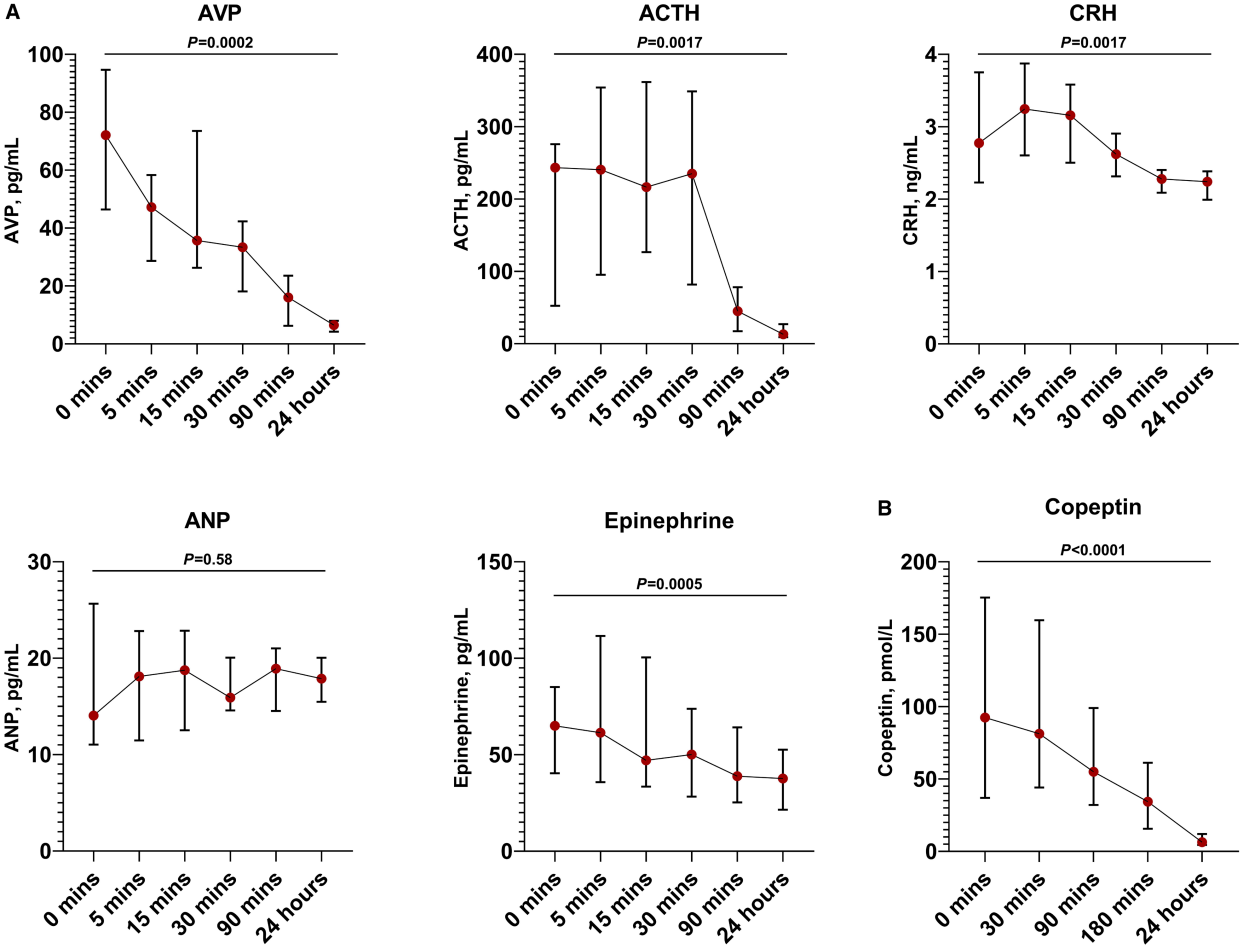
Plasma concentration of five hormones and copeptin from pre‐reperfusion to 24 hours post‐reperfusion. **A**, Median plasma concentrations of 5 hormones during acute ST‐segment–elevation myocardial infarction (STEMI) and reperfusion (n=13). **B**, Median plasma concentration of copeptin during STEMI and reperfusion in two cohorts (n=100 for 0 minutes, 30 minutes, and 24 hours; n=45 for 90 minutes; n=55 for 180 minutes). 0 minutes represents the value immediately before reperfusion with primary percutaneous coronary intervention, other time points are after reperfusion. ACTH indicates adrenocorticotropic hormone; ANP, atrial natriuretic peptide; AVP, arginine vasopressin; and CRH, corticotropin‐releasing hormone. Values at 0 minutes and 24 hours compared using the Wilcoxon matched‐pairs signed rank test. Error bars represent interquartile range.

Our study focused primarily on copeptin as a stable surrogate for AVP. However, in a subset (n=13) of the MRI cohort, we serially measured AVP itself, alongside other hormones possibly involved in the stress response to MI: adrenocorticotrophic hormone, corticotrophin‐releasing hormone, atrial natriuretic peptide, and epinephrine (Figure 1A). We found that both AVP and adrenocorticotrophic hormone were significantly higher acutely, falling by 24 hours (*P*=0.0002 and *P*=0.0017, respectively). AVP gradually declined at each time point, whereas adrenocorticotrophic hormone remained markedly elevated until falling substantially between 30 and 90 minutes. Corticotrophin‐releasing hormone and epinephrine were both significantly higher at baseline than 24 hours, although the magnitude of the change was much smaller than that seen with AVP and adrenocorticotrophic hormone. Atrial natriuretic peptide did not change significantly across time points. We then established how the concentration of copeptin, as the principal marker under investigation, changes during STEMI and reperfusion. Both our IMR and MRI cohorts had copeptin measured serially, starting before reperfusion. In the IMR cohort, this was measured again at 30 minutes, 180 minutes, and 24 hours after reperfusion. In the MRI cohort, time points were 30 minutes, 90 minutes, and 24 hours. These data show a clear trend of decreasing copeptin concentration over time after reperfusion, in keeping with the trend observed in AVP concentration. Median copeptin concentration before reperfusion was 92.5 pmol/L, falling to 6.4 pmol/L by 24 hours (Figure 1B). Copeptin levels did not differ between male and female subjects, but we did find a statistically significant weak to moderate correlation between age and copeptin levels at all time points except 180 minutes, the strongest of which was at 24 hours (r=0.352, *P*=0.001).

### Admission Copeptin Inversely Correlates With Admission Troponin

**Figure 2:**
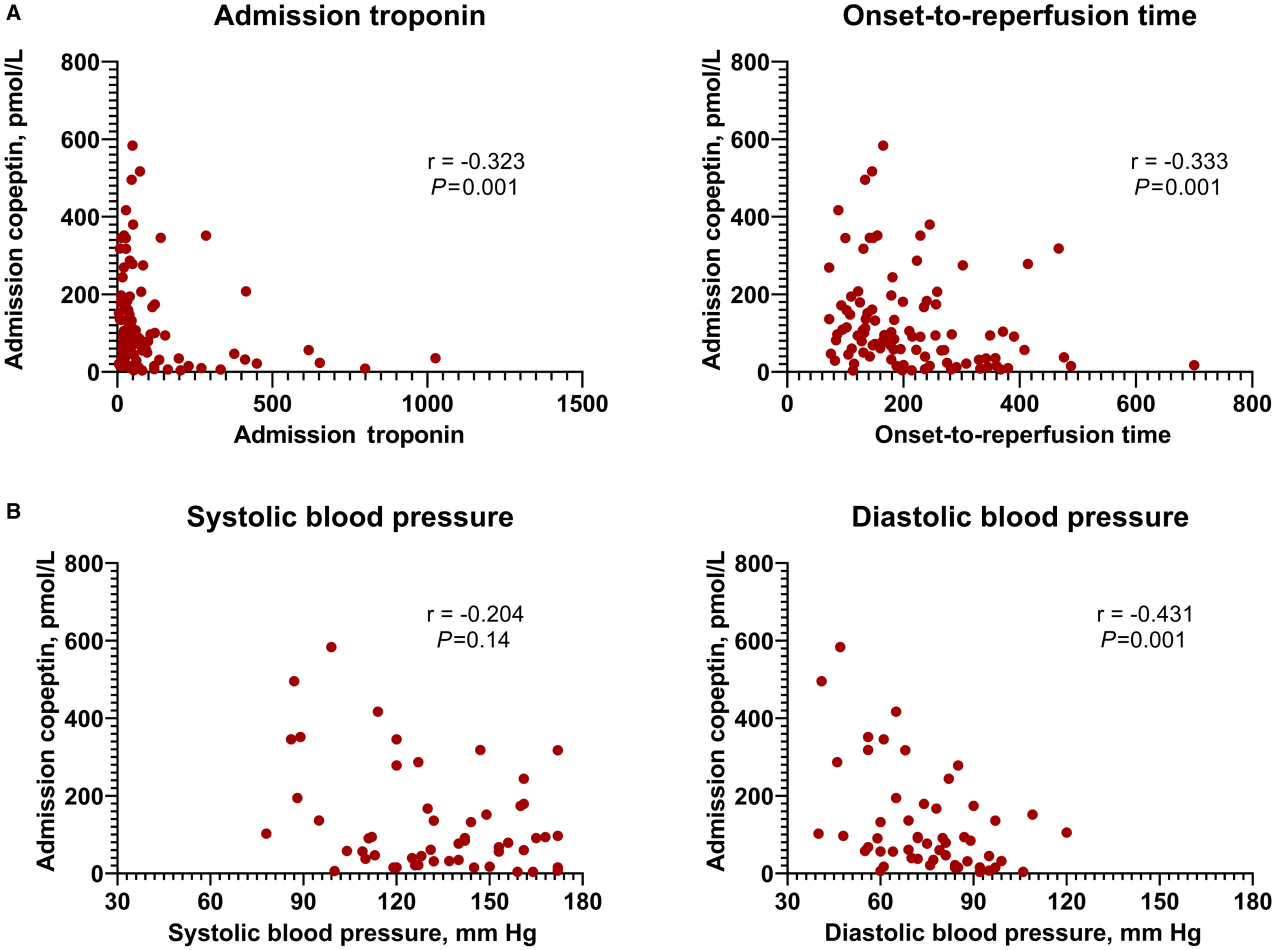
Variables associated with admission copeptin value. **A**, Relationship between admission copeptin concentration and admission serum troponin T and onset‐to‐reperfusion time (n=100). Both showed a negative correlation with admission copeptin concentration. **B**, Relationship between admission copeptin concentration and blood pressure (n=55). Only patients in the IMR cohort had admission blood pressure data available. Diastolic, but not systolic, blood pressure showed a significant negative correlation with admission copeptin concentration. Correlations determined with Spearman's rho. *P*<0.05 considered significant. IMR indicates index of microcirculatory resistance.

To help establish whether copeptin concentration falls in response to reperfusion of the culprit artery, or is already falling when the patient arrives in hospital, we looked at the relationship of admission copeptin with both admission troponin and the time between onset of chest pain and reperfusion (onset‐to‐reperfusion time). We found negative correlations between admission copeptin and both admission troponin (Spearman's r=−0.323, *P*=0.001) and onset‐to‐reperfusion time (r=−0.333, *P*=0.001; Figure 2A). These associations remained statistically significant when the 2 cohorts were analyzed separately (Figure S1). The relationship between copeptin and troponin shows a cluster of patients with both low troponin and low copeptin and a striking absence of patients with both high copeptin and high troponin. We hypothesize this is due to large troponins resulting from a longer period since the start of myocardial ischemia, by which time the copeptin has already fallen.

### Copeptin Release Is Associated With Diastolic Hypotension

A major function of AVP is blood pressure regulation, and we hypothesized that either systemic or myocardial hypoperfusion would influence admission copeptin concentration. We therefore assessed the correlation between arterial blood pressure and copeptin level in the IMR cohort (n=55). We found a moderate inverse correlation between admission copeptin concentration and admission diastolic blood pressure (DBP; r=−0.431, *P*=0.001), and a nonsignificant association with admission systolic blood pressure (r=−0.204, *P*=0.14; Figure 2B). As myocardial perfusion is determined by DBP, rather than systolic blood pressure, this supports the hypothesis that AVP is released in direct response to myocardial hypoperfusion.

### Coronary Microvascular Dysfunction on Invasive Testing Is Associated With Persistently Raised Copeptin at 24 Hours

In the 55 patients who underwent invasive testing of the coronary microcirculation, we measured copeptin before reperfusion and 30 minutes, 180 minutes, and 24 hours after reperfusion. IMR and CFR values were used both as continuous variables and grouped into low (IMR ≤40, n=33 and CFR <2, n=31) and high (IMR >40, n=22 and CFR ≥2, n=23) as previously described, with microvascular dysfunction indicated by high IMR or low CFR.

We found significant correlations between 24‐hour copeptin concentration and both high IMR (r=0.372, *P*=0.011) and low CFR (r=−0.467, *P*=0.001), both indicating coronary microvascular dysfunction. No association was seen between microvascular dysfunction and copeptin at earlier time points, likely due to the large effect of onset‐to‐reperfusion time on earlier copeptin concentrations.

**Figure 3:**
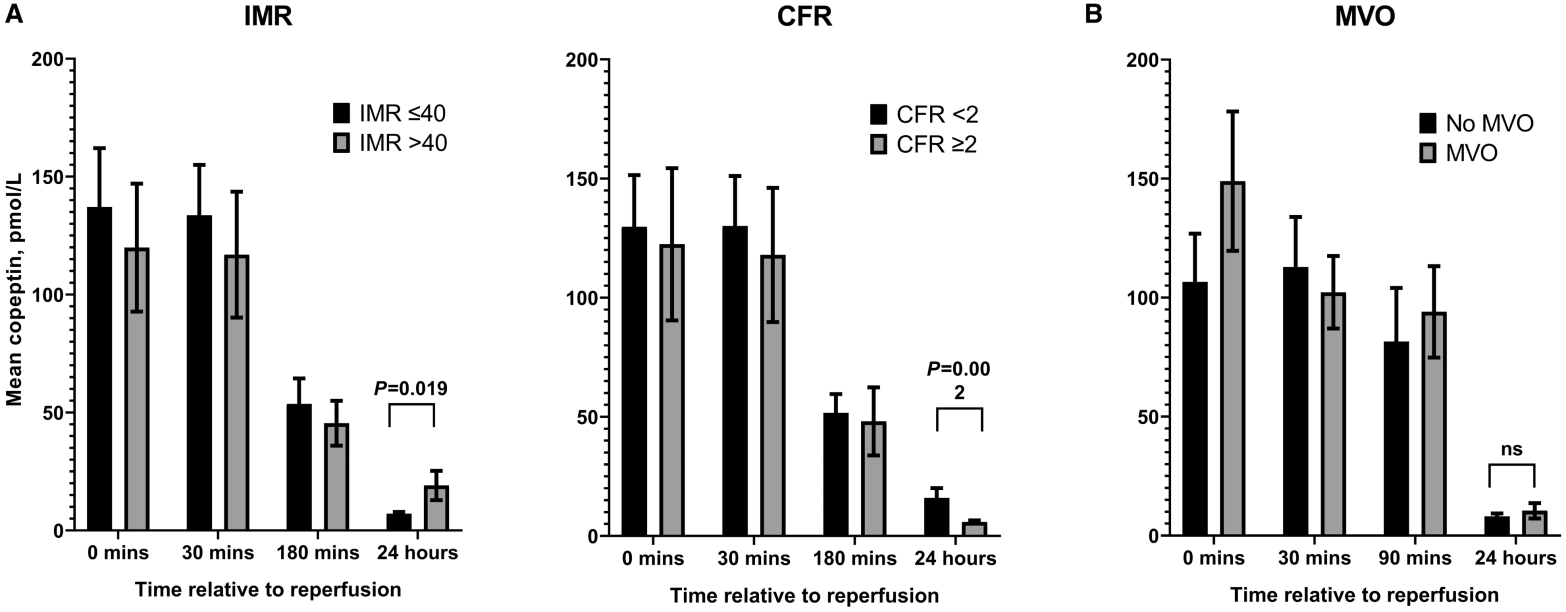
Copeptin concentration and objective measures of coronary microvascular dysfunction. **A**, Trend of copeptin before and after reperfusion in patients with STEMI, comparing those with evidence of coronary microvascular dysfunction (CMD) on invasive testing (IMR >40, n=22; CFR <2, n=23) with those without (IMR ≤40, n=33; CFR ≥2, n=31). Copeptin was significantly higher at 24 hours in patents with CMD. **B**, Trend of copeptin in similar patients, comparing those with microvascular obstruction (MVO) on cardiac magnetic resonance imaging (n=20) and those without (n=25). Hypothesis testing was with Mann–Whitney *U* test. *P*<0.05 was considered significant. CFR indicates coronary flow reserve; IMR, index of microcirculatory resistance; and STEMI, ST‐segment–elevation myocardial infarction.

Patients with an IMR >40, who have previously been shown to have worse outcomes after PPCI, had significantly higher mean copeptin concentration at 24 hours compared with those with lower IMR (19.1 versus 7.1 pmol/L, *P*=0.019). Patients with a CFR <2 also had significantly higher mean copeptin at 24 hours (15.9 versus 5.9 pmol/L, *P*=0.002; Figure 3A). These data suggest that persistent elevation of copeptin (and therefore AVP release) at 24 hours is associated with microvascular dysfunction.

### Copeptin Concentration Did Not Predict the Presence of Microvascular Obstruction, or Final Infarct Size, on CMR

Finally, we looked at the association between copeptin concentrations and noninvasive measures of coronary microvascular dysfunction, with both early (2–8 days, n=44) and late (3 months, n=43) CMR after STEMI. Early MVO is the primary radiological finding of microvascular dysfunction, and was present in 20 patients (45.5%) on early MRI. We found that patients with MVO on early CMR had a higher median copeptin concentration at baseline (102.0 pmol/L versus 68.7 pmol/L; Figure 3B), although this was not statistically significant (*P*=0.263). Copeptin concentration was not associated with any other findings on early CMR.

## DISCUSSION

CMD is a complex, incompletely understood phenomenon that is clearly linked to adverse outcomes after PPCI, with no targeted treatment currently available. This study has investigated the role of AVP, by measurement of its surrogate copeptin, in this process. We show that copeptin is released in response to myocardial ischemia and, as copeptin reaches greater concentrations in those with a low DBP, suggest it is a response to coronary hypoperfusion. In almost all patients it then falls rapidly, as shown by patients with high admission troponin, and longer onset‐to‐reperfusion times, having lower admission copeptin values. However, patients with persistently elevated copeptin at 24 hours were more likely to have coronary microvascular dysfunction on invasive assessment. AVP receptor antagonists may represent a novel therapeutic option in patients with STEMI and evidence of microvascular dysfunction.

### Copeptin Release After Myocardial Ischemia

The rapid release of copeptin after myocardial ischemia, and subsequent return to baseline levels within 24 hours, has been well established in previous studies.^24^, ^25^, ^26^ Whether this rapid fall is hastened by timely epicardial reperfusion, or would occur regardless, is unclear. Similar to Gu et al, we saw lower copeptin levels in patients with a longer duration of symptoms,^25^ which suggests that the fall in copeptin is not dependent on reperfusion. Previous evidence also suggests that more extensive ischemia leads to greater copeptin release. Copeptin release was less pronounced in patients with unstable angina as compared with STEMI.^27^ Moreover, during transcoronary alcohol ablation of septal hypertrophy, during which a small MI is induced, copeptin rises within 30 minutes but the peak value is considerably lower than found in patients with STEMI in this study.^28^ An innovative study by Árnadóttir et al, in which patients with normal coronary arteries were subjected to up to 90 seconds of ischemia through balloon occlusion of the left anterior descending artery, found that troponins, but not copeptin, were detectably raised after such a brief period of ischemia.^29^ This suggests that although copeptin rises very rapidly in response to substantial myocardial ischemia and necrosis, it does not rise in response to very minor, brief periods of myocardial ischemia. We also found that DBP inversely correlates with copeptin concentration and suggest this is due to myocardial hypoperfusion (as myocardial perfusion is governed by DBP) triggering copeptin release. This finding agrees with that reported by Vargas et al,^30^ who showed that lower DBP predicts higher copeptin concentration in 852 patients presenting with suspected MI. Direct release of copeptin from the myocardium is unlikely, as analysis of transcoronary gradient showed no difference in copeptin concentration in the aorta or coronary sinus in patients with acute MI.^31^ This suggests an alternative signaling pathway may be responsible for pituitary copeptin release in response to myocardial hypoperfusion.

### Association of 24‐Hour Copeptin Concentration and CMD

Early copeptin concentrations, as we have demonstrated, are greatly influenced by both time since the onset of ischemia and hemodynamic state. It is therefore not surprising that copeptin at these time points did not predict the presence or absence of CMD. At 24 hours, however, by which time copeptin has usually returned to near‐normal, elevated copeptin was associated with a raised IMR and a low CFR, both suggestive of CMD. CMD after STEMI involves several mechanisms, including impaired vasomotor function^32^ and vasospasm,^33^ and the link between AVP and CMD is complex. Although AVP has been reported to induce epicardial vasodilation after MI,^34^ it has also been shown to cause vasoconstriction, particularly of the microvasculature, in several studies.^35^, ^36^ Further, AVP can produce vasoconstriction of small coronary arteries sufficient to induce myocardial ischemia without affecting epicardial blood flow.^17^ Although the effects of AVP are attenuated during ischemia,^37^ they become enhanced when normoxia is restored,^38^ such as after reperfusion,^39^ and this may contribute to ischemia–reperfusion injury, a known mechanism for CMD after STEMI.^4^ In summary, AVP may contribute to CMD after MI through microvascular vasoconstriction, possibly exacerbated by the return of normoxia after reperfusion.

### Limitations

Our study has several important limitations. First, precise description of the effect of AVP on the coronary microvasculature lies beyond the scope of this observational study, and this should be further explored with a clinical trial involving an AVP‐modifying drug alongside invasive measurement of microvascular function. Second, our comparatively small sample of patients, the majority of whom were male, limits the generalizability of the results, and without long‐term follow‐up we do not know the clinical implications of the CMD we have observed. Further, we do not have data on the race of study participants in the MRI cohort, but the large majority of the IMR cohort were White, again possibly limiting the generalizability of our findings. A larger study is required to confirm our findings, and to explore the role of AVP antagonism in treating CMD following STEMI. We suggest this study should include a greater representation of women and minority groups than was possible here and be adequately powered to assess sex‐based differences.

## CONCLUSIONS

The pathophysiology of CMD after STEMI is complex, and we suggest that AVP release is an important mechanism. Current evidence suggests that therapies targeting coronary vasospasm after STEMI are ineffective,^33^ and only limited evidence of the benefit of vaptans is available from animal models.^40^ Given this study's findings, treatment with an AVP antagonist holds promise in reducing CMD after STEMI, and further investigation with a randomized clinical trial is warranted.

## Sources of Funding

This work was supported by a grant from the Newcastle upon Tyne Hospitals National Health Service Foundation Trust Charity (RES/0200/8073). Ioakim Spyridopoulos is supported by the British Heart Foundation (PG/23/11093 and PG/22/10788).

## Disclosures

Ioakim Spyridopoulos receives research grants from AstraZeneca (Cambridge, UK), Kancera (Stockholm, Sweden), and TA Science (New York, NY). The remaining authors have no disclosures to report.

## Supporting information

Figure S1
