# Supplementary material for: Arginine Vasopressin Plays a Role in Microvascular Dysfunction After ST‐Elevation Myocardial Infarction
Source: J Am Heart Assoc. 2023 Sep 8;12(18):e030473. doi: 10.1161/JAHA.123.030473 (PMC10547306; doi:10.1161/JAHA.123.030473)
Supplement: Supplementary file 1 — Figure S1 [file JAH3-12-e030473-s001.pdf]

SUPPLEMENTAL MATERIAL

Figure S1. Relationship between admission copeptin level and admission troponin T (left) and onset-to-reperfusion time (right) in each cohort.

IMR cohort, n=55

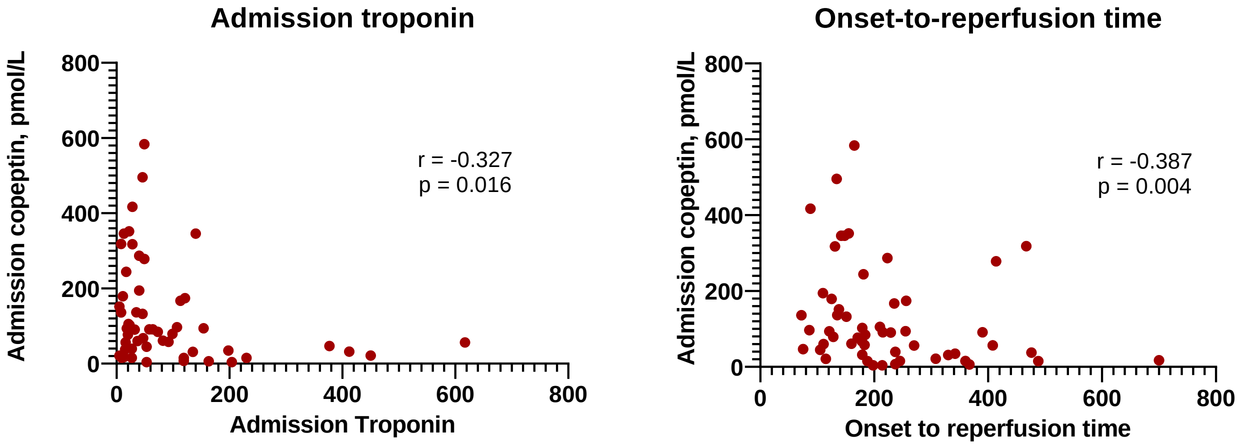

MRI cohort, n=45

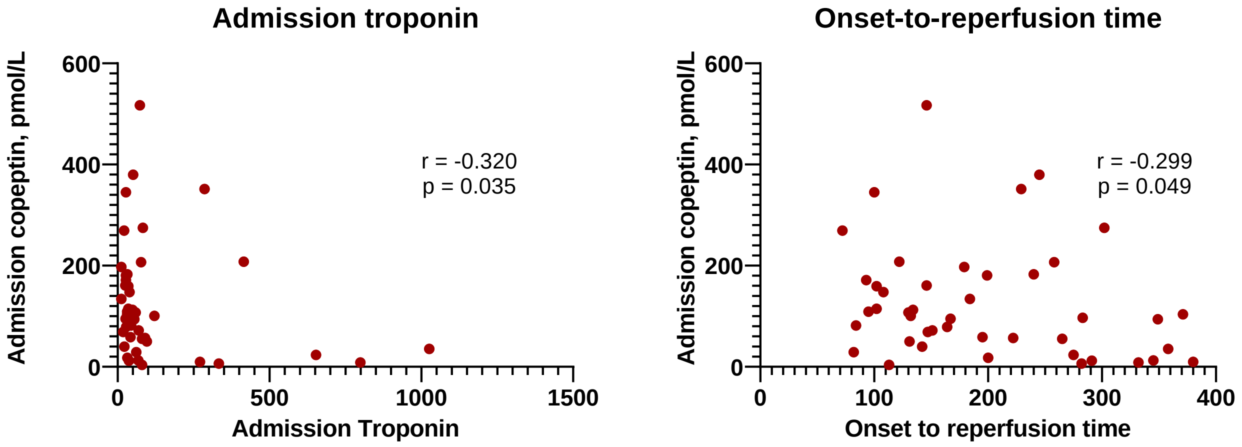

Correlations determined with Spearman’s rho. $p<0.05$ considered significant.
